# Evaluation of hyper-lipidaemia as a predictive marker for gestational diabetes among Indian women

**DOI:** 10.6026/9732063002001206

**Published:** 2024-10-31

**Authors:** Shivani Chitale, Sajaykumar Patil, Nitin S. Kshirsagar

**Affiliations:** 1 Department of Obstetrics and Gynecology, Krishna Institute of Medical Sciences, Karad - 415110, Maharashtra, India

**Keywords:** Gestational diabetes mellitus, 12-14 weeks, hyperlipidemia levels, pregnancy, oral glucose tolerance test

## Abstract

Gestational diabetes mellitus (GDM) is an impaired capacity to metabolize carbohydrates throughout pregnancy; its occurrence is increasing in around 20-27% globally. Therefore, it is of interest to know the hyperlipidemia levels at 12-14 weeks with GDM. 100 patients were assessed using history, general, systemic, antenatal and routine examination along with follow-up at 20 weeks for Oral Glucose Tolerance Test (OGTT). Data shows that triglyceride, total cholesterol and hyperlipidemia levels showed significant predictors of development of GDM as the p value was <0.05. Although gestational diabetes mellitus is linked to surgical deliveries and foetal problems such as macrosomia yet more studies are recommended to validate the data.

## Background:

Gestational diabetes mellitus (GDM) is a disorder that arises or is first recognized during pregnancy and is characterized by an impaired capacity to metabolize carbohydrates [1]. They also found that, this illness may be addressed by dietary modifications or insulin treatment, both of which are effective in managing the condition and its relevance resides in the fact that both present & future generation's risk of developing DM increases [1]. In another study author found that, the prevalence of GDM was growing globally accounts at least 20-27% of all pregnancies [2]. They also found the prevalence rate in India after analyzing & comparing between rural area and urban area that, it was around 1.7% -13.2% and 4.6% -14% [3].

In the latter stages of pregnancy, women who have diabetes are more likely to have high blood pressure [4, 5]. Hyperlipidemia is frequently observed during the latter stages of pregnancy, as it is considered a necessary physiological mechanism to supply the fetus with fuel and nutrients [6]. There is no particular biochemical test that can accurately forecast the likelihood of developing GDM, except from the diagnostic blood sugar tests such as oral glucose challenge test (OGCT) and OGTT, as both of these tests are considered to be diagnostic tests [7]. There are significant physiological changes that occur in the pregnant woman and these changes are necessary in order to fulfill the requirements of both the mother and the fetus [8]. The rise in triglyceride levels during the onset of pregnancy appears to be linked to insulin resistance and, consequently, gestational diabetes, a significant pregnancy complication [9]. According to Mudd *et al.* there is a link between higher levels of total cholesterol (TC), low-density lipoprotein-cholesterol (LDL-C) and triglycerides (TG) during the 15-27 week period of pregnancy and an increased likelihood of suffering a spontaneous premature delivery. The association persists throughout the duration of the pregnancy [10]. A clear connection between gestational dyslipidemia and adverse birth weight outcomes is shown [11]. Therefore, it is of interest to report hyperlipidemia at 12-14 weeks as a prognostic indicator of GDM and to examine the maternal and perinatal outcomes for these individuals.

## Materials and Methods:

The current hospital based observational study was conducted at obstetrics and gynaecology department in a tertiary care hospital with a total of 100 in number patients we included out of 426 ANC women, starting from June 2022 ending to November 2023 with 18 months in total after ethical committee approval. The data was collected with the help of interview in person each patient which includes detailed clinical history, general, systemic and antenatal examination with routine investigation, to ensure their comfort and have their full cooperation by the investigator. Venous blood sample was taken from patient between 12 to 14 weeks gestation and was sent for complete lipid profile. The patients were asked for follow-up at 20 weeks of gestation for OGTT & other necessary ANC care.

## Measurement of lipid:

In this, serum triglyceride was estimated by glycerol-3-phosphate oxidase - phenol - aminophenazone (GPO-PAP) method using RANDOX kit. The triglycerides are measured following enzymatic hydrolysis using lipases. The indicator is produced by the catalytic action of peroxidase on a mixture of hydrogen peroxide, 4-aminophenazone and 4-chlorophenol, resulting in the formation of a Quinoneimine compound. Total cholesterol levels were determined using the enzymatic end point approach using the RANDOX kit. Cholesterol is measured following enzymatic hydrolysis and oxidation. The indicator Quinoneimine is synthesized by combining hydrogen peroxide with 4-aminoantipyrine in the presence of phenol and peroxidase. HDL-C estimation was performed using the immuneturbidometric approach using the RANDOX kit. The magnitude of the quinine imine dye generated is closely correlated with the content of HDL cholesterol when assessed at a wavelength of 600 nm. This assay employs a rate technique and a calibration based on a single point. LDL-C estimation was calculated by using Friedewald equation.

LDL-C = [Total Cholesterol] - [HDL-C] - [Triglyceride/5], the factor [Triglyceride/5] is an estimate of the VLDL-C concentration.

## Inclusion criteria:

All ANC patients coming to OPD

## Exclusion criteria:

[1] BMI >40 kg/m^2^

[2] Undergone bariatric surgery

[3] H/O dyslipidaemia

[4] Patients k/c/o DM

## Statistical analysis:

Study used SPSS version 2.6 Microsoft excel 2021. The qualitative data was shown using frequency and percentage. The association between qualitative factors was evaluated using a Chi-Square test. The quantitative data was expressed using the mean value plus or minus the standard deviation. The quantitative data between the two groups was analyzed using an unpaired t-test if the data passed the 'Normality test' and by the Mann-Whitney Test if the data failed the 'Normality test'.

## Results:

Table 1 shows that, out of 426 cases, 100 patients were of dyslipidemia (DL) with 23.4%. Among these 100 patients, 23 developed GDM in DL cases (10.4%). On the other hand, 326 patients were N-DL while 34 developed GDM in N-DL cases (23%). Table 2 shows that, among various lipid abnormalities, out of 100 patients, raised LDL & VLDL were seen in 51% and 52% cases while low HDL was observed in 49% cases and raised triglyceride and total cholesterol levels were observed in 24% and 25% cases respectively. Table 3 shows that, maximum numbers of cases were seen between 26-30 years with 158 for N-DL & 45 for DL. Thus, showed no statistically significant difference between the groups as the p value was 0.06. Table 4 shows that, maximum numbers of cases were seen with 76 with and 251 without dyslipidemia. Thus showed no difference between the groups as the p value was 0.94. Table 5 shows that, DL was observed in 67 patients (24.3%), DL with DM was observed in 33 patients (22.0%) while only DM was observed in 117 (78.0%). Thus, data showed that non-significant difference as the p value was 0.643. Table 6 shows that, mean gestation age was 38.44 & 37.89 weeks for DL & N-DL. Thus showed no significant difference as the p value was 0.11. Table 7 shows that, prevalence of GDM was 23% among DL cases and 10.4% for N-DL. This showed a significant difference as the p value was <0.01. Table 8 shows that, mean triglyceride levels (113.4 vs 95.8 mg%; p<0.01) and cholesterol levels (158.02 vs 144.24 mg%; p<0.01) were significantly higher and HDL levels (50.25 vs 53.79 mg%; p<0.01) were significantly lower in DM cases. Table 9 shows that, assisted vaginal delivery (26.1% vs 7.8%) and LSCS (52.2% vs 36.4%) was observed to be significantly higher among cases of GDM as the p value was <0.01.

Table 10 shows that, among maternal complications, incidence of fever (39.1% vs 10.4%) and UTI (17.4% vs 2.6%) was significantly higher among cases of GDM as the p value was <0.01 and 0.04 while non-significant for sepsis (8.7% vs 1.3%) and wound gape (13.0% vs 2.6%) respectively as the p value was 0.15 & 0.10. Table 11 shows that, mean APGAR at 1 and 5 minutes was not statistically comparable between cases of GDM and controls as the p value was 0.48 and 0.85 respectively. Table 12 shows that, mean birth weight was significantly higher among cases of GDM (3.08 vs 2.66 Kg as the p value was <0.01. Table 13 shows that, among FC, incidence of macrosomia (26.1% vs 0%), hypoglycemia(8.7% vs 0%) and RDS (8.7% vs 1.3%) was significantly higher among cases of GDM as the p value was <0.01, 0.04 and 0.08 respectively. Table 14 shows that, NICU admission was observed in 17.4% cases of GDM as compared to 5.2% cases on non-GDM. Thus, showed no significant difference as the p value was 0.079. Table 15 shows that, triglyceride, total cholesterol and HDL levels were observed to be significant predictors of development of GDM (p<0.05). At a cut-off of TG> 110 mg%, sensitivity and specificity was 72.2% and d 62.2%. At a cut-off of TC> 140 mg%, sensitivity and specificity was 79.4% and 69.5%. At a cut-off of HDL< 45 mg%, sensitivity and specificity was 77.8% and 68.3%, respectively.

## Discussion:

In our study we have observed that, out of 26 patients, 100 patients were of dyslipidemia, giving a prevalence of 23.4% for dyslipidemia in pregnancy. Among various lipid abnormalities, raised LDL and VLDL were seen in 51% and 52% cases while low HDL was observed in 49% cases. Raised triglyceride and total cholesterol levels were observed in 24% and 25% cases respectively. Baseline parameters like age, gestation age, BMI and family history of diabetes were comparable between cases with and without dyslipidemia. According to a study conducted in 2023, it was found that a quarter of pregnant women who had lipid testing in their first trimester had abnormal results. Adding a lipid panel to routine prenatal screenings during the first trimester can help identify familial hypercholesterolemia (FH) in women. As revealed by the study, this illness might affect both the mother and the kid [12]. Based on a study conducted by Herrera-Martinez *et al.* it was found that the prevalence of dyslipidemia varied between 27% and 86%. Both groups showed rates of 9.9% and 61.9% respectively, indicating that decreased plasma HDL levels were the predominant lipid issue. Following that, there was a prevalence of hypertriglyceridemia at a rate of 36.5% and a rate of 9.6% [13]. In a study conducted by Saliu *et al.* it was discovered that a significant number of pregnant women experienced various lipid abnormalities during the second trimester of pregnancy. Specifically, 69.6% of the women had dyslipidemia, 19.6% had hypercholesterolemia, 36.6% had hypertriglyceridemia, 18.8% had elevated levels of low-density lipoprotein and 49.1% had reduced levels of high-density lipoprotein in their serum lipid profiles. During the third trimester, there was a significant increase in the values of these parameters, reaching 91.8%, 54.1%, 75.3%, 40.0% and 62.4%, respectively [14].

Guo *et al.* showed that there is a significant increase in the risk of gestational diabetes mellitus (GDM) with each unit rise in the TyG index. This finding was determined through logistic regression analysis. Even when considering other factors, this increase in risk remained significant. When it comes to predicting GDM, the TyG index outperformed other factors with an impressive area under the curve (AUC) value of 0.641 and a 95% confidence interval (CI) of 0.61-0.671 in the receiver operating characteristic (ROC) curve. After careful analysis, it was found that the optimal threshold value is 8.890, exhibiting a sensitivity and specificity of 0.617 each [15]. In a study conducted by Wang *et al.* the researchers aimed to evaluate the significance of lipid profiles and fasting glucose levels during early pregnancy in predicting the development of GDM. Receiver operator characteristics analysis was utilized in the analysis. According to the findings, certain factors like lipid profiles during early pregnancy, including cholesterol, triacylglycerols, ratios of LDL-C/HDL-C and ratios of TG/HDL-C, may serve as potential indicators for predicting GDM [16]. In not more recent study conducted by Jacobson JD and colleagues, they revealed that patients with gestational diabetes mellitus (GDM) had a higher incidence of caesarean sections (LSCS) due to problems such as cephalopelvic disproportion (CPD) and fetal macrosmia. This was determined by examining a large sample of patients [17]. In our study we found that, assisted vaginal delivery (26.1% vs 7.8%) and LSCS (52.2% vs 36.4%) was observed to be significantly higher among cases of gestational diabetes. Among maternal complications, incidence of fever (39.1% vs 10.4%) and UTI (17.4% vs 2.6%) was significantly higher among cases of gestational diabetes. Moreover, mean birth weight was significantly higher among cases of gestational diabetes (3.08 vs 2.66 Kg; p<0.01). Mean APGAR at 1 and 5 minutes was comparable between cases of gestational diabetes and controls (p>0.05). Among Fetal complications, incidence of macrosomia (26.1% vs 0%) and hypoglycemia (8.7% vs 0%) was significantly higher among cases of gestational diabetes. NICU admission was observed in 17.4% cases of gestational diabetes as compared to 5.2% cases on non-GDM (p-0.079). Li *et al.* conducted a study that revealed notable disparities in birth weight and macrosomia across the two groups [18]. Nanda *et al.* discovered that individuals with gestational diabetes had a higher incidence of foetal problems such as macrosomia, shoulder dystocia, stillbirth, hypoglycemia, congenital malformations and trauma after delivery [19]. A research conducted by Balaji *et al.* found that 9.9% of women with gestational diabetes mellitus (GDM) experienced macrosomia [20]. Dyslipidemia, characterized by low HDL-C levels and an elevated triglycerides/HDL-C ratio, is observed with greater frequency in children born to mothers with GDM. This condition may adversely affect the progression of atherosclerosis during childhood. Enhancing the screening and management of dyslipidemia in pediatric populations is essential, particularly for those whose mothers experienced GDM [21].

## Conclusion:

Data shows that GDM is linked to increased rates of surgical deliveries and foetal problems such as macrosomia. It is advisable to assess the lipid profile during pregnancy in order to identify patients that are at risk. The effective management of lipids throughout the second trimester will decrease the occurrence of GDM and serve as a viable approach to enhance clinical outcomes in these women at high risk.

## Figures and Tables

**Table 1:** Baseline data distribution

**Data**	**N**
Total cases	426
Dyslipidemia	100 (23.4%)
Non-Dyslipidemia	326
GDM	57 (13.4%)
GDM in Dyslipidemia Cases	23 (10.4%)
GDM in Non- Dyslipidemia Cases	34 (23%)

**Table 2:** DL distribution

**Type of Dyslipidemia**	**N (n-100)**	**%**
Raised TG	24	24.00%
Raised TC	25	25.00%
Low HDL	49	49.00%
Raised LDL	51	51.00%
Raised VLDL	52	52.00%

**Table 3:** Age distribution

**Age in years**	**Dyslipidemia**		**Total**
	**No**	**Yes**	
< =20	8	6	14
	2.50%	6.00%	3.30%
21-25	131	32	163
	40.20%	32.00%	38.30%
26-30	158	45	203
	48.50%	45.00%	47.70%
> 30	29	17	46
	8.90%	17.00%	10.80%
Total	326	100	426
	100.00%	100.00%	100.00%
p-value-0.06			

**Table 4:** BMI distribution

**BMI (Kg/m^2^)**	**Dyslipidemia**		**Total**
	**No**	**Yes**	
<18.5	0	0	0
	0.00%	0.00%	0.00%
18.5-25	32	11	43
	9.80%	11.00%	10.10%
25.1-30	251	76	327
	77.00%	76.00%	76.80%
> 30	43	13	56
	13.20%	13.00%	13.10%
Total	326	100	426
	100.00%	100.00%	100.00%
p-value-0.94			

**Table 5:** F/H of DM

**F/ H of DM**	**Dyslipidemia**		**Total**
	**No**	**Yes**	
No	209	67	276
	75.70%	24.30%	100.00%
Yes	117	33	150
	78.00%	22.00%	100.00%
Total	326	100	426
	76.50%	23.50%	100.00%
p-value-0.643			

**Table 6:** DL with mean GA

**Variables**	**Dyslipidemia**	**N**	**Mean**	**SD**	**p- value**
Gestation Age (GA) (weeks)	No	326	37.89	1.39	0.11
	Yes	100	38.44	1.4	

**Table 7:** GDM

**GDM**	**Dyslipidemia**		**Total**
	**No**	**Yes**	
Yes	34	23	57
	10.40%	23.00%	13.40%
No	292	77	369
	89.60%	77.00%	86.60%
Total	326	100	426
	100.00%	100.00%	100.00%
p-value<0.01			

**Table 8:** Lipid profile

**Lipid profile (mg %)**	**Group**	**N**	**Mean**	**SD**	**p- value**
TG	GDM	57	113.4	37.45	< 0.01
	Non-GDM	369	95.8	28.68	
TC	GDM	57	158.02	33.89	< 0.01
	Non-GDM	369	144.24	30.58	
HDL	GDM	57	50.25	5.95	< 0.01
	Non-GDM	369	53.79	6.26	
LDL	GDM	57	75.41	21.79	0.497
	Non-GDM	369	77.71	24.12	
VLDL	GDM	57	25.44	7.38	0.601
	Non-GDM	369	26.1	9.03	
LDL/ HDL	GDM	57	1.51	0.4	0.712
	Non-GDM	369	1.48	0.55	

**Table 9:** MOD

**Mode of Delivery (MOD)**	**Dyslipidemia Group**		**Total**
	**GDM**	**Non-GDM**	
Vaginal	5	43	48
	21.70%	55.80%	48.00%
Assisted Vaginal	6	6	12
	26.10%	7.80%	12.00%
LSCS	12	28	40
	52.20%	36.40%	40.00%
Total	23	77	100
	100.00%	100.00%	100.00%
p-value <0.01			

**Table 10:** MC

**Maternal Complications (MC)**	**Dyslipidemia Group**		**p-value**
	**GDM**	**Non-GDM**	
Fever	9	8	< 0.01
	39.10%	10.40%	
UTI	4	2	0.04
	17.40%	2.60%	
Sepsis	2	1	0.15
	8.70%	1.30%	
Wound Gape	3	2	0.1
	13.00%	2.60%	

**Table 11:** APGAR score

**APGAR**	**Dyslipidemia Group**	**N**	**Mean**	**SD**	**p- value**
at 1 min	GDM	23	7.39	0.5	0.48
	Non-GDM	77	7.48	0.55	
at 5 min	GDM	23	8.7	0.47	0.85
	Non-GDM	77	8.68	0.47	

**Table 12:** BW

**Variable**	**Dyslipidemia Group**	**N**	**Mean**	**SD**	**p- value**
Birth Weight (kg)	GDM	23	3.08	0.81	< 0.01
(BW)	Non-GDM	77	2.66	0.43	

**Table 13:** FC

**Fetal Complications (FC)**	**Dyslipidemia Group**		**p-value**
	**GDM**	**Non-GDM**	
Macrosomia	6	0	< 0.01
	26.10%	0.00%	
Hyperbilirubinemia	0	2	1
	0.00%	2.60%	
Hypocalcemia	0	1	1
	0.00%	1.30%	
Hypoglycemia	2	0	0.04
	8.70%	0.00%	
RDS	2	2	0.08
	8.70%	1.30%	

**Table 14:** NICU

**NICU**	**Group**		**Total**
	**GDM**	**Non-GDM**	
No	19	73	92
	82.60%	94.80%	92.00%
Yes	4	4	8
	17.40%	5.20%	8.00%
Total	23	77	100
	100.00%	100.00%	100.00%
p-value-0.079			

**Table 15:** ROC curve analysis

**Ideal Cut-off**	**Sensitivity**	**Specificity**	**Accuracy**
TGs> 110 mg%	72.20%	62.20%	67.20%
TC> 140 mg%	79.40%	69.50%	74.50%
